# Fish Scale-Derived Scaffolds for Culturing Human Corneal Endothelial Cells

**DOI:** 10.1155/2018/8146834

**Published:** 2018-04-29

**Authors:** Mohit Parekh, Bert Van den Bogerd, Nadia Zakaria, Diego Ponzin, Stefano Ferrari

**Affiliations:** ^1^International Center for Ocular Physiopathology, Fondazione Banca degli Occhi del Veneto Onlus, Venice, Italy; ^2^Department of Molecular Medicine, School of Biomedicine, University of Padova, Padova, Italy; ^3^Ophthalmology, Visual Optics and Visual Rehabilitation, Translational Neurosciences, Faculty of Medicine, University of Antwerp, Antwerp, Belgium; ^4^The Centre for Cell Therapy and Regenerative Medicine, Antwerp University Hospital, Antwerp, Belgium

## Abstract

**Purpose:**

To investigate the biocompatibility of fish scale-derived scaffolds (FSS) with primary human corneal endothelial cells (HCEnCs).

**Methods:**

HCEnCs were isolated from 30 donor corneas in a donor-matched study and plated in precoated Lab-Tek slides (*n* = 15) and FSS (*n* = 15). Cell morphology, proliferation/migration, and glucose uptake were studied (*n* = 30). Hoechst, ethidium homodimer, and calcein AM (HEC) staining was performed to determine viability and toxicity (*n* = 6). The cell surface area was calculated based on calcein AM staining. HCEnCs were stained for ZO-1 (*n* = 6) to detect tight junctions and to measure cell morphology; Ki-67 (*n* = 6) to measure proliferating cells; and vinculin to quantify focal adhesions (*n* = 6). The formation of de novo extracellular matrix was analyzed using histology (*n* = 6).

**Results:**

HCEnCs attach and grow faster on Lab-Tek slides compared to the undulating topography of the FSS. At day 11, HCEnCs on Lab-Tek slide grew 100% confluent, while FSS was only 65% confluent (*p* = 0.0883), with no significant difference in glucose uptake between the two (*p* = 0.5181) (2.2 *μ*g/mL in Lab-Tek versus 2.05 *μ*g/mL in FSS). HEC staining showed no toxicity. The surface area of the cells in Lab-Tek was 409.1 *μ*m^2^ compared to 452.2 *μ*m^2^ on FSS, which was not significant (*p* = 0.5325). ZO-1 showed the presence of tight junctions in both conditions; however, hexagonality was higher (74% in Lab-Tek versus 45% in FSS; *p* = 0.0006) with significantly less polymorphic cells on Lab-Tek slides (8% in Lab-Tek versus 16% in FSS; *p* = 0.0041). Proliferative cells were detected in both conditions (4.6% in Lab-Tek versus 4.2% in FSS; *p* = 0.5922). Vinculin expression was marginally higher in HCEnCs cultured on Lab-Tek (234 versus 199 focal adhesions; *p* = 0.0507). Histological analysis did not show the formation of a basement membrane.

**Conclusions:**

HCEnCs cultured on precoated FSS form a monolayer, displaying correct morphology, cytocompatibility, and absence of toxicity. FSS needs further modification in terms of structure and surface chemistry before considering it as a potential carrier for cultured HCEnCs.

## 1. Introduction

The human cornea is the outermost, transparent tissue of the eye. It is the principal refractive element of the visual system, and its function depends mainly on its optical clarity. Human corneal endothelial cells (HCEnCs) are responsible for maintaining this transparency through a pump-and-leak mechanism [1]. To do so, this leaky barrier of hexagonally shaped cells allows passive diffusion of nutrients flowing from the anterior chamber to the corneal stroma and epithelium but simultaneously averts corneal edema by pumping excessive fluid back to the anterior chamber.

Due to a mitotic arrest *in vivo* after birth, the number of endothelial cells decreases throughout life [2]. However, this decay can dramatically be accelerated by trauma or several diseases. If the overall number of HCEnCs drops below a certain threshold of less than 500 cells/mm^2^, irreversible edema eventually arises, leading to an opaque cornea.

The only available treatment currently is corneal endothelial transplantation, termed endothelial keratoplasty (EK). In 2016, nearly 40% of donated corneas distributed by US eye banks were transplanted to treat endothelial dysfunction. Although EK has a high success rate in terms of visual rehabilitation and postoperative visual outcome, transplantations are often restricted by a shortage of corneal donor tissue [3].

In order to overcome this scarcity, alternative therapeutic approaches such as ex vivo expansion of HCEnCs are under investigation to enable HCEnCs transplantation as cell sheets or cell suspension [4–7]. Once HCEnCs from one donor eye can successfully be expanded, we would finally be able to overcome the current 1 : 1 ratio where one donor cornea is used to treat a single patient. Consequently, waiting lists would shorten significantly. In case of the cell sheet transplantation strategy, a scaffold is required which will act as a mechanical support (i.e., a surrogate basement membrane) that can sustain cell proliferation and phenotype. Multiple scaffolds have been reported as candidate membranes, and among these options, three different categories can be identified: (i) biological, (ii) synthetic, and (iii) biosynthetic substrates [5].

In 2010, Lin et al. proposed an oxygen- and glucose-permeable collagen scaffold derived from decalcified fish scales (Tilapia; *Oreochromis mossambicus*) that can be used in corneal regeneration [8]. Until now, preliminary *in vitro* studies have shown cytocompatibility of corneal epithelial cells on these heterogeneously patterned, biological scaffolds [9]. Its architectural features have been suggested as an important characteristic for corneal epithelial cell migration and growth. Moreover, its transparency and availability, that is, roughly 200 scales from one fish, make it an attractive biocompatible material for the generation of corneal epithelial cell grafts. Additional *in vivo* studies performed on rats and rabbits have demonstrated its potential as a deep anterior lamellar keratoplasty (DALK) alternative or to seal perforated corneas, respectively [10].

Although fish scale-derived collagen scaffolds (FSS) have been identified as a potential scaffold for ocular surface reconstruction, its potential to support HCEnC cultures has not yet been explored. If FSS enable early attachment and growth of HCEnCs, they could serve as a potential carrier in tissue engineering corneal endothelial grafts. This paper therefore investigates the potential of a fish scale-derived collagen scaffold to support the attachment and proliferation of primary HCEnCs. In addition, we evaluate its effect on cell viability and preservation of key proteins (i.e., ZO-1 tight junctions), which are characteristics for the HCEnC barrier formation.

## 2. Materials and Methods

### 2.1. Ethical Statement

Human donor corneas [*n* = 30, fifteen pairs] were collected from the Veneto Eye Bank Foundation (FBOV) with informed consent from the donors' next of kin to be used for research. The methods followed the tenets of the Declaration of Helsinki, and the tissues were used under the laws of Centro Nazionale di Trapianti. The corneas were unsuitable for transplantation due to their low endothelial cell counts (<2200 cells/mm^2^) and thereby qualified as research grade, with no known additional complications or contraindications. All tissues were preserved in tissue culture medium at 31°C prior to use for experiments.

### 2.2. Donor Characteristics

The average donor age was 60.75 (±14.55) [range: 45–75] years, and the male : female ratio was 10 : 5. The postmortem time to the preservation of the corneas was 16.54 (±5.89) hours. The tissues were preserved in tissue culture medium for 31.25 (±6.78) days prior to isolation of the cells. Average endothelial cell density (ECD) before isolation was found to be 1965 (±202.83) cells/mm^2^ in corneas obtained for Lab-Tek and 1970 (±191.76) cells/mm^2^ for FSS. For the experiments, one cornea was used for 2 Lab-Tek wells of 0.7 cm^2^ each and the other cornea from the same donor (donor-matched study) was used for 1 fish scale of 13 mm diameter with a surface area of 1.32 cm^2^. The corneas did not show any dead cells determined using trypan blue staining before plating.

### 2.3. Processing and Characteristics of Fish Scale Scaffolds

Tilapia fish scales were cleaned and acellularized using previously reported methods [8–11]. Briefly, the harvested fish scales were rinsed in distilled water and decellularized according to a four-step detergent and enzymatic processing, involving a stepwise protease, surfactant, and DNase and RNAse treatment, followed by a final surfactant treatment [12]. Acetic acid was used to increase the porosity of the scaffolds, followed by decalcification with nitric acid [8]. The resulting acellularized fish scales were rinsed extensively, stored, and transported in sterilized phosphate-buffered saline (PBS). FSS were then shipped to the FBOV labs from Body Organ Biomedical Corporation (Taipei, Taiwan) as acellularized scaffolds.

Each FSS was 13 mm in diameter with an average thickness of 100–120 *μ*m. Tensile stress was 12.68 MPa (±9.53), and Young's modulus was 56.4 MPa (±21.91) with an elongation of 24.72 (±5.65)%. Water holding capacity of the FSS was 82% (±3.0) with an initial transparency of 92.67% within the visual spectrum (380–780 nm) as recorded by Body Organ Biomedical Corporation prior to shipping the FSS to FBOV labs. The surface topography of fish scales was observed using anterior iVue Optical Coherence Tomography (OCT) (OptoVue, California, USA).

### 2.4. Endothelial Cell Count and Donor Characteristics

Cell death (%) was determined prior to isolation, using 0.25% trypan blue (TB) (Thermo Fisher Scientific, New York, USA). Approximately 100 *μ*L of TB was topically applied to stain the endothelial cells for 20 seconds followed by washing with 1x PBS. Trypan blue-positive cells and ECD of three random areas were counted by two operators before enzymatic digestion of the cells using an in-built eyepiece reticule (10 × 10 1 mm^2^ boxes) for inverted microscopy (Axiovert, Zeiss, Germany). Donor characteristics of the 15 donors (30 corneas in total) were obtained from the FBOV database to determine age, gender, postmortem time to preservation, cause of death, and duration of preservation.

### 2.5. Formulation of the Medium for Cell Culture

Proliferation medium composition was similar to that described earlier [13–16] and was a mixture of Ham/F12 (Sigma-Aldrich, St. Louis, Missouri, USA), M199 (Sigma-Aldrich, St. Louis, Missouri, USA), 5% FBS (Sigma-Aldrich, St. Louis, Missouri, USA), 1% ascorbic acid (Sigma-Aldrich, St. Louis, Missouri, USA), 0.5% insulin transferrin selenium (Thermo Fisher Scientific, Waltham, Massachusetts, USA), 10 ng/mL recombinant human FGF basic (Thermo Fisher Scientific, Waltham, Massachusetts, USA), 10 *μ*M Rho-associated coiled-coil protein kinase (ROCK) inhibitor Y-27632 (Miltenyi Biotec, Bergisch Gladbach, Germany), and 1% PenStrep (Thermo Fisher Scientific, Waltham, Massachusetts, USA) [13].

### 2.6. Primary HCEnC Isolation

**Figure 1:**
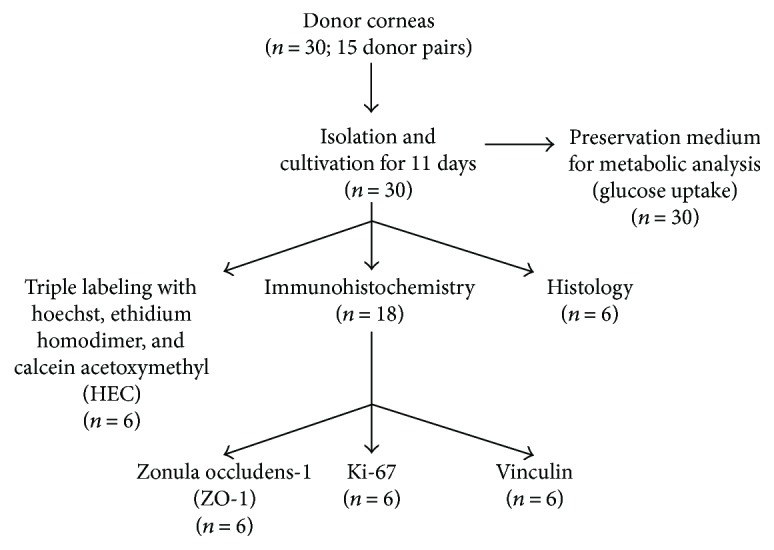
An overview of the performed experiments with the amount of corneal donor pairs used in each group.

HCEnCs were isolated from research grade donor corneas using a peel-and-digest method similar to previously published methods, [7, 13–15] with limited modifications. Firstly, the corneas [*n* = 30] were washed in sterile PBS and Descemet's membrane with endothelium was dissected with a fine forceps, similar to the stripping technique used for Descemet's membrane endothelial keratoplasty (DMEK). Secondly, the excised pieces were incubated in 2 mg/mL collagenase type 1 (Thermo Fisher Scientific, Rochester, NY, USA) solution for 2-3 hours at 31°C, 5% CO_2_. Once Descemet's membrane was digested, the solution was centrifuged for 5 minutes at 1000 rpm. The supernatant was removed, and the cells were resuspended in TrypLE Express (1x) for 10 minutes at 37°C, (Life Technologies, Monza, Italy) to obtain single cell suspension suitable for seeding. An overview of the performed experiments can be seen in Figure 1.

### 2.7. Cell Culture and Morphological Analysis

Lab-Tek II chamber slides (8 × 0.7 cm^2^ culture area) from Thermo Fisher Scientific (Rochester, NY, USA) and FSS (13 mm diameter) were used for culturing cells of each donor pair (*n* = 30; fifteen pairs). Per donor, two chambers of Lab-Tek slides and one FSS were coated with FNC coating mix (US Biological Life Sciences, Salem, Massachusetts, USA) for at least 30 minutes at 37°C. When seeding primary cells (passage 0), the seeding density for the Lab-Tek slide (control group) was 180,645 (±19,265) cells, which was divided between the two wells of Lab-Tek slides and 181,120 (±18,215) cells were plated on a single FSS. The cell suspension was added in a small volume on the concave side of the FSS and on the Lab-Tek slide and incubated at 37°C for 20 minutes allowing the cells to settle. An additional volume of proliferation medium was added once the cells showed attachment. Cultures were monitored and refreshed every alternate day until confluence. The percentage of confluency was manually measured by the area of outgrowth using a built-in reticule inside the eye-piece of the microscope, that is, number of boxes filled with the cells of a 10 × 10 reticule of 1 mm^2^ each.

### 2.8. Glucose Uptake of the Cultured HCEnCs for Functional and Metabolic Analysis

Glucose uptake was determined from preserved medium that was stored at −20°C (*n* = 30) every alternate day. Quantitative analysis was performed using the D-Glucose HK kit (Megazyme International Ireland Ltd., Bray Business Park, Bray, County Wicklow, Ireland). With this, the amount of glucose utilized by the HCEnCs was determined, allowing the evaluation of metabolic activity over time. Positive controls in this experiment were cells grown on Lab-Tek slides, while negative controls were samples containing culture medium without cells.

### 2.9. Hoechst, Ethidium Homodimer, and Calcein Acetoxymethyl (AM) (HEC) Staining to Determine Live and Dead Cells

Cell cultures from three donors at confluence (day 11) were washed with PBS prior to the assay. The control sample consisted of isolated Descemet's membrane, with intentionally damaged areas to induce cell death. The HEC mastermix consisted of 5 *μ*L of Hoechst 33342 (blue) (Thermo Fisher Scientific, Rochester, NY, USA), 4 *μ*L of ethidium homodimer EthD-1 (red), and 2 *μ*L calcein AM (green) (Live/Dead viability/cytotoxicity kit, Thermo Fisher Scientific, Rochester, NY, USA) mixed in 1 mL of 1x PBS [17]. 100 *μ*L of the final solution was directly added on the cell cultures and control samples and incubated at room temperature (RT) in the dark for 45 minutes. The control sample was prepared for a flat mount and covered with mounting medium. HEC staining was viewed at 50x and 100x magnifications of the LSM 510-metalaser scanning microscope (Zeiss, Milan, Italy).

### 2.10. Immunostaining for Tight Junctions, Proliferation, and Focal Adhesions

Cells from three donors at confluence (day 11) were used for each study. The cells were washed with PBS and fixed in 4% paraformaldehyde (PFA) at RT for 30 minutes and permeabilized with 0.5% Triton X-100 in PBS for 30 minutes. After blocking with 2% goat serum for 2 hours at RT, the tissues were incubated overnight at 4°C with primary antibodies anti-ZO-1, 1 : 200 (ZO1-1A12, Thermo Fisher Scientific, Rochester, NY, USA); anti-Ki-67, 1 : 200 (MIB-1, Milan, Italy); and anti-vinculin, anti-collagen VI, and anti-laminin I, 1 : 200 (Abcam, Cambridge, Massachusetts, USA). The samples were incubated with goat anti-mouse fluorescein isothiocyanate- (FITC-) conjugated secondary antibody in 20% goat serum for 2 hours at RT. After each step, the cells were washed 3 times with 1x PBS and covered with mounting medium and cover slips. Samples were examined with the LSM 510-metalaser scanning microscope (Zeiss, Milan, Italy).

### 2.11. Histological Analysis

Histology was performed using cells cultured on FSS from three different donors. After day 11, upon cellular confluence on the control condition, the FSS were washed with 1x PBS for 5 minutes and fixed in 4% PFA overnight followed by consecutive washing with sucrose solution at 7.5%, 15%, and 30% for 15 minutes each. After final washing, tissues were embedded in Optimal Cutting Temperature medium for microtome sectioning. Periodic acid-Schiff (PAS) staining was performed on all samples, and sections were viewed at 200x and 400x magnifications. A normal human cornea was used as a control. Anti-collagen VI (ab118955) [1 : 200] and anti-laminin I (ab11575) [1 : 200] (Abcam, Cambridge, UK) antibodies were used to check the presence of extracellular matrix with Draq 5 (Thermo Fisher Scientific) as a nuclear counterstain. The immunostaining procedure remained the same as above.

### 2.12. Measurement and Statistical Analysis

ImageJ software bundled with Java 1.8.0_101 version (National Institutes of Health, USA) was used for image analysis and quantification purposes. Three microscopic fields were selected for each evaluation (one central and two mid-peripheral). The cell surface area was determined on 10 cells per condition at 100x magnification using Calcein AM and analyzed with “analyze particles” with size limits of 150–10,000 *μ*m^2^ considering there were no background signal and large cell clusters. For ZO-1, the area was selected and using predefined commands in Macros for converting the image to overlay masks, the total number of cells was automatically counted whereas the hexagonal cells and polymorphic cells were counted based on the cell structure in the particular area (with 6 borders) at 100x magnification. The macros was designed particularly for this study to obtain results by simply inserting the algorithm in the ImageJ analysis. The particles were analyzed at 100x magnification using outline option, and watershed was applied if necessary for Ki-67-positive cells. For vinculin, focal adhesion points of ten cells per sample were counted and the average number of focal adhesions was recorded for analysis using binary masks. Data are expressed as the mean ± standard deviation (SD). A nonparametric Wilcoxon test for paired data using SAS statistical software was employed to check the statistical significance between different conditions where *p* < 0.05 was deemed significant.

## 3. Results

### 3.1. Characteristics of the Fish Scale Scaffolds

**Figure 2:**
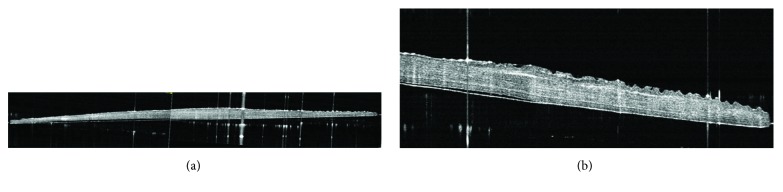
Optical coherence tomography (OCT) imaging demonstrated (a) nonuniform thickness of the fish scale scaffold and (b) the extent of grooves present on the edges of the scaffold.

On a montage of multiple OCT images, the scales did not appear to have a uniform thickness but were thinner peripherally (Figure 2(a)). It was also possible to detect the surface topography of the FSS with its distinct valleys and ridges (Figure 2(b)).

### 3.2. Morphology, Confluency, and Glucose Uptake

**Figure 3:**
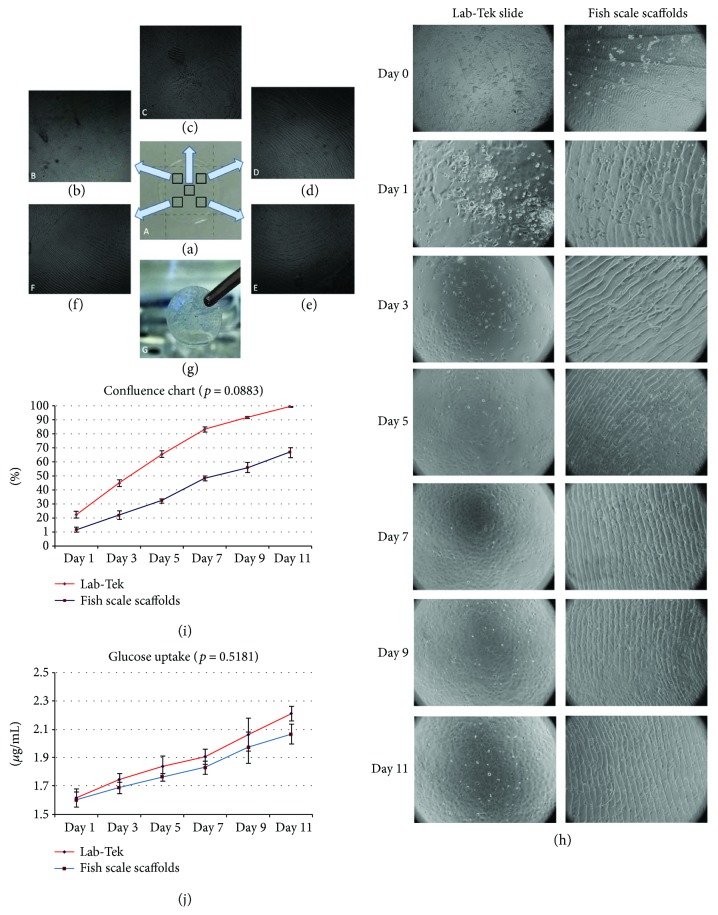
(a) FSS showed transparency, and when observed in bright field of the confocal microscope, (b) it showed narrow ridges and valleys. (c) Central FSS was rough but was relatively a flat surface compared to the other areas. (d and f) Second and third quadrants, respectively, showing broad ridges and valleys. (e) Fourth quadrant showing arc strokes. (g) Although the transparency of FSS after HCEnC culture was not objectively determined, the scaffold did not show any damage or high opacity when observed subjectively. (h) Differences in morphology between Lab-Tek and FSS grown HCEnCs at 40x magnification (for day 0, to appreciate the number of cells that were plated) and 100x magnifications on the rest of the days of culture. Most of the cells adhered and showed outgrowth on broad ridges (quadrants 2 and 3) and in the central FSS. (i) Growth rate of the cells in Lab-Tek was higher compared to that on FSS. 100% confluency was observed in Lab-Tek slides whereas around 65% confluency was observed in FSS at day 11 (*p* = 0.0883). (j) Glucose uptake was recorded in Lab-Tek-grown cells compared to fish scale scaffolds and showed active metabolism of cells without any significant difference between the two conditions (*p* = 0.5181). The data are expressed as the mean ± SD. FSS: fish scale scaffold; HCEnC: human corneal endothelial cell. Scale: (B–D) 100x magnification.

FSS displayed a nonhomogeneous surface architecture consisting of broad and narrow troughs and ridges, spokes, and a central flat region (Figures 3(a)–3(f)). Transparency of the FSS remained unchanged when observed before and after HCEnC culture (Figures 3(a) and 3(g)), as observed subjectively. The cells showed improved adherence on areas with broad ridges, but also centrally, where the surface was flatter (Figure 3(h)). The growth rate of the cells in Lab-Tek was marginally higher compared to that on FSS. At day 11, cells covered 65% of the FSS, while the controls were completely confluent (*p* = 0.0883) (Figure 3(i)). Average glucose uptake was not different for FSS and control conditions (*p* = 0.5181), that is, 2.2 *μ*g/mL in Lab-Tek versus 2.05 *μ*g/mL in FSS (Figure 3(j)) at day 11.

### 3.3. Cell Viability and Cell Area on FSS

**Figure 4:**
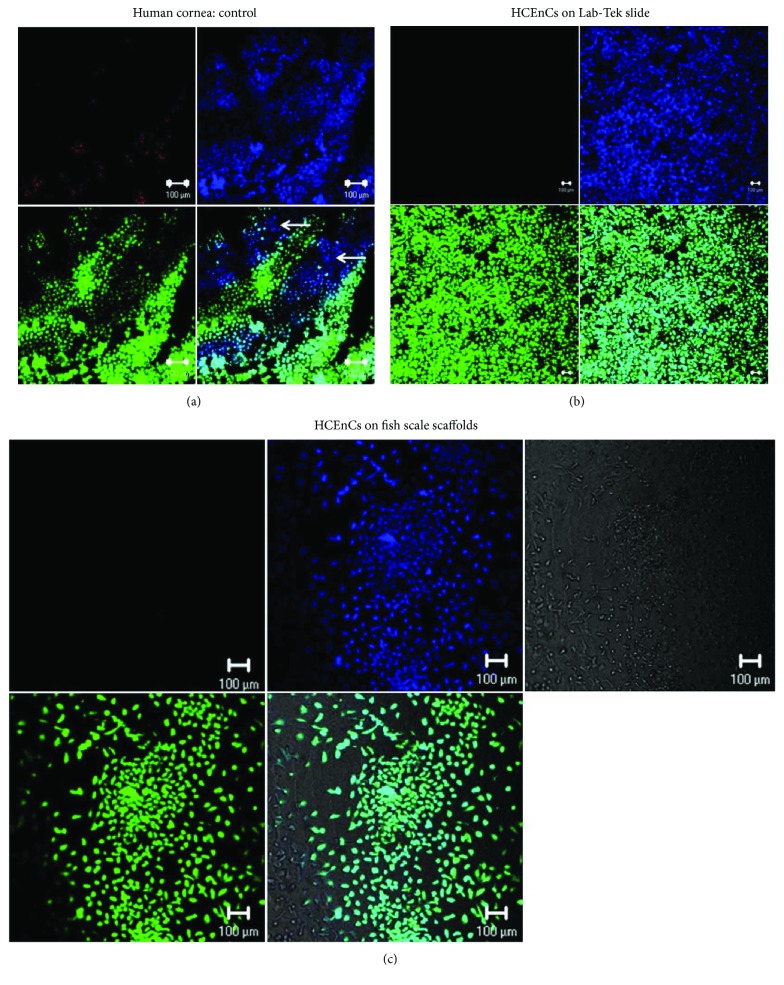
Triple staining using HEC to determine live/dead cells. (a) Control cornea to show the presence of dead (red), live (green), and dying cells (blue without green marked with white arrows). Cell nuclei are shown in blue cells. (b) HCEnCs from old donor corneas cultured on Lab-Tek showed high viability and confluency without any dead cells. (c) Relatively lesser amount of cells was observed when HCEnCs were cultured on FSS. There were no dead cells observed on FSS too, but again, most of the cells were cultured in quadrants 2 and 3. HCEnCs: human corneal endothelial cell; FSS: fish scale scaffold.

Triple labelling with HEC showed the dead cells in red, the nuclei in blue, and live cells in green. A human donor cornea used as a control to demonstrate the HEC staining showed dead cells (red), nuclei (blue), live cells (green), dying cells (blue without green), and merge (Figure 4(a)). Only a few apoptotic cells were observed manually by counting blue cells (nuclei) without green (cytoplasm) marked as white arrows (Figure 4(a)). HCEnCs on Lab-Tek slides showed high viability as shown in Figure 4(b). In compliance with the confluency data, HEC staining also showed that the cells were approximately 60% confluent (Figure 4(c)) on the FSS with almost 100% viability in both conditions. The cell area was determined on 10 cells per condition using calcein AM staining and ImageJ analysis. Average values of the cell area in the Lab-Tek slide was found to be 409.1 *μ*m^2^ (±169.1) compared to 452.2 *μ*m^2^ (±131.1) on FSS, which was not found to be statistically significant (*p* = 0.5325).

### 3.4. Immunostaining

**Figure 5:**
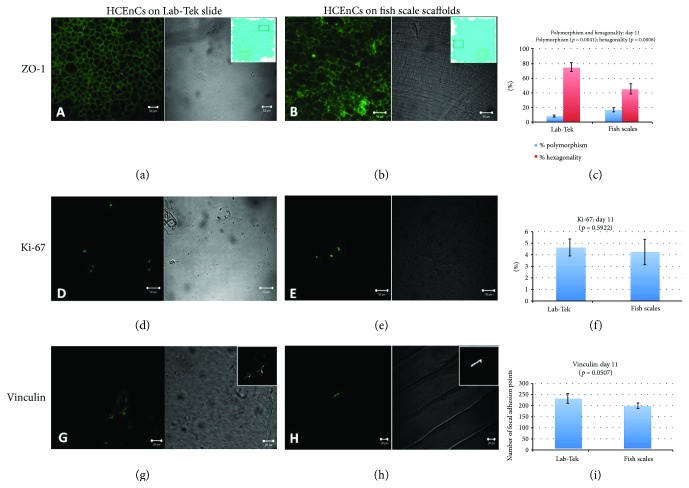
Expression of different proteins in cultured HCEnCs on Lab-Tek and FSS. ZO-1 staining for intracellular tight junctions to determine polymorphism and hexagonality of the cells in (a) Lab-Tek and (b) in FSS. Figure inserts show the overlay masks of the expression for calculation of hexagonality and polymorphism (polymegathism and pleomorphism). (c) High percentage of polymorphism (*p* = 0.0041) and low percentage of hexagonality (*p* = 0.006) were observed when HCEnCs were cultured on FSS compared to Lab-tek slides. (d) Proliferative cells did not differ when the HCEnCs were cultured in (e) Lab-Tek and FSS and (f) did not show statistical significance (*p* = 0.5922). (g) Vinculin as a focal adhesion marker showed a higher number of focal adhesions when HCEnCs were cultured on Lab-Tek compared to that on (h) FSS which was (i) marginally significant (*p* = 0.0507). Figure inserts show the amount of vinculin expressed (number of focal adhesions) which was counted using binary images. HCEnC: human corneal endothelial cell; FSS: fish scale scaffold.

ZO-1 tight junction protein was expressed in HCEnCs cultivated on both FSS (Figure 5(a)) and control (Figure 5(b)). HCEnCs on Lab-Tek showed 8.1% (±1.5) polymorphism with 74.7% (±6.1) hexagonality whereas those cultured on FSS showed 16.7% (±2.8) polymorphism and 45.1% (±6.8) hexagonality (Figure 5(c)). HCEnCs cultured on Lab-Tek slides showed significantly less polymorphism (*p* = 0.0041) and a higher percentage of hexagonal cells (*p* = 0.0006). It was also noted that the cells cultured in the central region of the FSS, which has a flatter topography, displayed a relatively better hexagon morphology as compared to peripheral regions.

HCEnCs cultured on Lab-Tek slides showed 4.6% (±0.7) Ki-67-positive cells (Figure 5(d)) compared to 4.2% (±1.1) on FSS (Figure 5(e)) indicating no significant difference (*p* = 0.5922) (Figure 5(f)).

HCEnCs cultured on Lab-Tek with FNC coating mix showed an average of 233.5 (±22.6) number of focal adhesions (Figure 5(g)) per cell (average of 10 cells counted in three microscopic fields) compared to 199.7 (±12.1) number of focal adhesions from FSS (Figure 5(h)) which was nearly reaching statistical significance than on Lab-Tek (*p* = 0.0507) (Figure 5(i)) at day 11. Initial investigative experiments showed that coating of the FSS was crucial for HCEnC attachment (data not shown).

### 3.5. Histological Analysis

**Figure 6:**
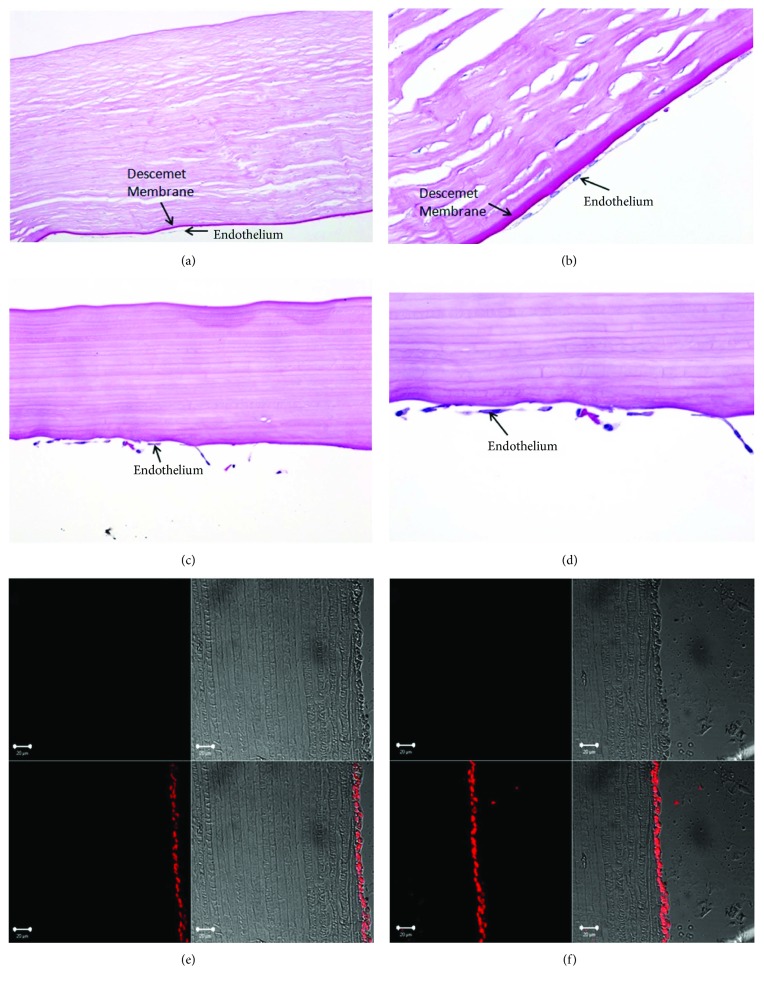
Histological analysis using periodic acid-Schiff staining. (a) A control human cornea showing the presence of Descemet's membrane and endothelial cells using PAS staining at 100x and (b) 400x. (c) FSS showing monolayer of endothelial cells at 200x magnification and (d) at 400x magnification. Histology did not show any clear evidence or development of Descemet's membrane on FSS by the HCEnCs but showed a monolayer of cells attached in most of the areas. (e) Collagen VI and (f) laminin as the extracellular markers did not express on the cells at day 11. Nucleus can be seen in red marked with Draq 5. FSS: fish scale scaffold; HCEnC: human corneal endothelial cell.

On whole mount control sections, periodic acid-Schiff (PAS) staining showed all corneal cell layers including Descemet's membrane and endothelium (Figures 6(a) and 6(b)). PAS staining showed that HCEnCs grew as a monolayer on FSS (Figure 6(c)). At day 11, the cells did not show the presence of their own basement membrane but only uniform distribution of corneal endothelial cells on the FSS (Figure 6(d)). This was further confirmed using collagen VI and laminin markers. No expression of collagen VI (Figure 6(e)) or laminin (Figure 6(f)) was observed on the FSS; however, Draq 5 showed the presence of cell nuclei on the FSS.

## 4. Discussion

One option to reduce global donor corneal shortage is to expand the HCEnCs from a single cornea into multiple transplantable sheets. However, these sheets require a carrier for transporting the cultured cells for transplantation. Development of a scaffold for culturing and transplanting expanded HCEnCs would thereby create composite grafts similar to current Descemet's stripping automated endothelial keratoplasty (DSAEK) procedures. Here, the donor endothelium with a residual layer of donor corneal stroma is inserted into the recipient's cornea using a tissue glider. It automatically unfolds when inserted in the anterior chamber after which the donor stromal tissue attaches to the acceptor stroma. It was observed that the FSS were flexible enough to be folded and unrolled automatically without breaking, similar to the DSAEK grafts. The FSS is primarily built of collagen type I, similar to the corneal stroma, so the attachment of the scaffold is expected to happen similarly. Also, transparency of FSS was acceptable and it did not degrade *in vivo* in any of the previously published studies [8–10].

Fish scales are inherently calcified; however, decalcification increases their transparency and degree of flexibility, thus improving its properties as a corneal scaffold. The question whether recalcification may occur following transplantation is difficult to ascertain *in vitro*. Only long-term animal studies could give a clear answer about the possibility of calcium precipitation on the FSS. However, animal studies using the FSS as a stromal implant for 3 weeks reported no such phenomenon, which was confirmed in another rabbit study over a period of 6 months [11, 18].

The scaffold was proved to be nontoxic and supported endothelial cell proliferation with the absence of dead/dying cells. HCEnCs adhered over the irregular substrate; however, we clearly observed regional differences in cellular proliferation. Cells showed better attachment on flatter areas over the narrow and higher ridges. In the more central regions and broad ridges, the morphology of the cells was similar to that of the control, whereas cells in the irregular regions showed a more stretched phenotype. We assume that the initial difficulty to attach on the irregular surface and the dimensions of the ridges further impedes the migration of the cells and thereby affects the formation of a confluent monolayer. These results corroborate with the findings published by Rizwan et al. [19] in which they point out that one of the limiting factors which affects monolayer formation was the spacing of artificial guttae, basement membrane excrescences that are characteristics for Fuch's endothelial dystrophy. When the spacing was too narrow, cells were not able to form a monolayer, whereas this did occur at a broader spacing. We had similar observations on the FSS, where cells preferentially attached to the broader ridges rather than the narrow spaces, also described in Table 1.

In this study, the immunocytochemical staining was carried out on day 11 as the HCEnCs in the control condition reached 100% confluence. When cells become too confluent, their expression pattern can change; hence, the staining was performed for the sake of identical conditions. Representative images of cell cultures were taken every alternate day. Through measuring the surface area of colonies on calibrated reticule, we objectively quantified the growth of endothelial cells by means of confluency. Traditional proliferation assays are based on cell metabolism and use a nonfluorescent dye that is added to a cell culture, which is then converted to a fluorescent dye; if metabolized, this signal then correlates with a certain number of cells. However, we did not know whether the metabolisms of cells grown on Lab-Tek and FSS are equivalent. If cells on one substrate metabolize at a higher rate, the amount of converted dye will not correlate to the corresponding number of cells. By measuring the glucose uptake and confluency separately, we confirmed this discrepancy. While the cells grown on Lab-Tek slides had become 90% confluent by day 9, they were only 50% confluent on the fish scales. However, glucose uptake was not significantly different, indicating a higher degree of (glucose) metabolism in cells grown on both, Lab-Tek and FSS. With distinct rates of metabolism, conversion-based proliferation assays therefore could not be used reliably in this study.

The cell surface area was not found to be significantly different between the two conditions. ZO-1, a tight junction protein associated with cell-cell interaction and one of the hallmarks of a HCEnC monolayer, is expressed appropriately in both conditions. This staining also allowed us to assess cell morphology, which revealed a higher degree of pleomorphism for HCEnCs when grown on FSS. Although cell areas were not significantly different and both the conditions were suitable for obtaining cell growth, the results highlight that similar cell area does not necessarily mean that the cells are hexagonal, which is an important parameter for HCEnC culture.

Vinculin, a membrane-cytoskeletal protein present in focal adhesions, is involved in the linkage of integrin adhesion molecules to the actin cytoskeleton. Staining for this protein allowed us to quantify the degree of cellular attachment with the substrate at a given time. Similar to observations with the light microscope, vinculin expression and thus attachment were higher on the Lab-Tek slides than on the FSS. As the control and FSS conditions are coated similarly, with collagen I and fibronectin, it is more likely that the surface topography is the main factor that adversely affects adhesion. Theoretically, the positive surface charge of the Lab-Tek, mimicking poly-L-lysine, could support the attachment to the slides even more. Similar to other research groups, we supplemented our proliferative medium with ROCK inhibitor since studies have reported that inhibition of ROCK signalling enhances the attachment of HCEnCs, hypothetically through the upregulation of vinculin [20, 21].

During initial investigative experiments, we saw that cells did not attach without the FNC coating. With the additional coating of the FSS, cells could produce their integrins more rapidly, accelerating the concomitant focal adhesion formation and ECM secretion. After 2 weeks of culture, sectional PAS staining showed that these cells formed a monolayer and did not stratify. There was no detection of extracellular matrix deposition at this time point. However, ECM deposition by HCEnCs *in vivo* is also very low, with the thickness of Descemet's membrane in the elderly being reported to be around 16 *μ*m [22, 23]. The difference between FSS and human corneal Descemet's membrane is listed in Table 2.

Apart from attachment, HCEnC proliferation on FSS was higher in control than in FSS. We did not observe a difference in metabolic activity or Ki67 staining, further supporting the noncytotoxic nature of the FSS and ability to sustain cellular proliferation to a certain degree.

The domain of tissue engineering is growing with the developments of cytocompatible and biodegradable materials. However, culture of HCEnCs and maintaining them on a scaffold still remain challenging. The FSS have a potential in terms of culturing challenging cell types like primary HCEnCs and, once decalcified, make a suitable corneal substitute. However, in many aspects, our control conditions on plastic performed better than the FSS. Our FSS were surface modified using ECM proteins (fibronectin and collagen I coating mix), so the inherent capacity of the FSS for cellular adhesion is still debatable, but we do show adequate attachment and proliferation once coated. Hence, we conclude that the fish scale-derived scaffold in its current form may not be ideal for the development of tissue-engineered corneal endothelial constructs. However, this study can certainly aid the further development of the FSS substrate, or other scaffolds, towards one that is specifically suitable for HCEnC cultures. Further modification of the substrate, such as surface polishing to remove the irregular topography, overall thinning of the scaffold, and incorporation of functional groups such as fibronectin, could drastically improve its geometric and physical characteristics while also enhancing cell-matrix interactions, in order to develop the ideal endothelial cell carrier.

## 5. Conclusion

The affordable nature and wide availability of this biological scaffold may attract further research into FSS as a robust tissue engineering scaffold. However, although FSS could have a promising future post-modification, as suggested, this model will have to undergo regulatory compliances similar to that of advanced therapy medicinal product (ATMP). This study is a proof of concept for culturing HCEnCs on FSS. While possessing attractive properties and cytocompatibility with primary corneal endothelial cells, additional refinement would be desired before testing in an animal model for cultivated endothelial transplantation [28].

## Figures and Tables

**Table 1:** Different dimensions of guttae reported in the article of Rizwan et al., both their literature searches and own measurements derived from retroillumination photography. In addition, we listed dimensions of the broad ridges of the FSS.

	Rizwan et al. [19]		Fish scale (broad ridges)
	Patient data	Literature	
Spacing	38 *μ*m	3–240 *μ*m	30 *μ*m
Width	20 *μ*m	6–70 *μ*m	3.4 *μ*m
Height	3.5 *μ*m	3–25 *μ*m	5.7 *μ*m

**Table 2:** Properties of the FSS compared to human corneal Descemet's membrane.

	Descemet's membrane	Fish scale scaffold
Diameter (mm)	10	13
Thickness (*μ*m)	8–35 [24, 25]	100–120
Tensile stress (MPa)	1.72 (±0.19) [26]	12.68 (±9.53)
Young's modulus (MPa)	0.050 ± 0.02 [27]	56.4 (±21.91)
Transparency (%)	>98 [24]	92.67
